# Risk of reintroduction of vaccine-preventable diseases in the state of São Paulo, Brazil

**DOI:** 10.11606/s1518-8787.2026060007062

**Published:** 2026-02-23

**Authors:** José Elisomar Silva de Santana, Maria Lígia Bacciotte Ramos Nerger, Tatiana Lang D’ Agostini, Joyce Ribeiro da Conceição Santos, Sheila Aparecida Ferreira Lachtim, Camilla Stephane Oliveira Silva, Thales Philipe Rodrigues da Silva, Fernanda Penido Matozinhos

**Affiliations:** I Coordenadoria de Controle de Doenças. Centro de Vigilância Epidemiológica "Prof. Alexandre Vranjac". Divisão de Imunização. São Paulo, SP, Brasil; II Universidade Federal de São Paulo. Escola Paulista de Enfermagem. São Paulo, SP, Brasil; III Universidade de São Paulo. Escola de Enfermagem. São Paulo, SP, Brasil; IV Universidade Federal de Minas Gerais. Escola de Enfermagem. Belo Horizonte, MG, Brasil; V Universidade Federal de Minas Gerais. Escola de Enfermagem. Departamento de Enfermagem Materno-Infantil e Saúde Pública. Belo Horizonte, MG, Brasil

**Keywords:** Vaccination Coverage, Risk Management, Environmental Monitoring, Disaster Surveillance

## Abstract

**OBJECTIVE:**

To analyze the risk classification of transmission of vaccine-preventable diseases in the municipalities of the state of São Paulo (SP) and to assess the influence of population size on the risk of transmission of vaccine-preventable diseases (RTDI) before and after the implementation of Microplanning for High-Quality Vaccination Activities.

**METHODS:**

This is an epidemiological study with an ecological design, conducted using secondary data on vaccination coverage for nine immunobiologicals recommended in the immunization schedule for children under two years of age. It also examines dropout rates for multi-dose vaccines within the same age group in the state of São Paulo, covering the years 2022 and 2023. Following the calculation of all indicators, municipalities were classified according to their risk of transmission for vaccine-preventable diseases.

**RESULTS:**

Analyses of vaccination coverage indicators, dropout rates, homogeneity of vaccination coverage among vaccines for children under two years of age, and the RTDI indicator for children aged one year or younger revealed that, in 2022, the human rotavirus vaccine had the highest proportion of municipalities reaching the coverage target (46%), followed by the meningococcal vaccine (39%). In 2023, the human rotavirus vaccine maintained the highest proportion of municipalities reaching the target (68%), followed by the pneumococcal vaccine (57%). Regarding the RTDI, in large municipalities, high risk fell from 100% to 91.2%, while in medium-sized municipalities there was a reduction from 90.8% to 66.7%, and in small municipalities, from 68.3% to 56%.

**CONCLUSION:**

Challenges remain within the state regarding the RTDI classification for vaccination, particularly in medium-sized and large municipalities. The use of immunization indicators at the municipal level can be highly effective for proposing health interventions aimed at increasing vaccination coverage.

## INTRODUCTION

The National Immunization Program (PNI) of Brazil, created in 1973, is responsible for the implementation of the National Immunization Policy and aims to reduce, control, and eliminate the transmission of vaccine-preventable diseases. On the international stage, Brazil's PNI is recognized worldwide for its broad vaccination schedule, available for all life stages, and for reducing the incidence and mortality of vaccine-preventable diseases, as well as for the eradication of some of them^1,2^. With regard to the history of the São Paulo State Immunization Program, immunization actions in the state have been recorded since 1962, prior to the creation of the PNI, when the first statewide vaccination campaign against poliomyelitis took place. In 1968, the first Technical Guideline of the Immunization Program of the São Paulo State Health Department was published.

The World Health Organization (WHO) emphasizes that vaccination coverage (VC) rates should be at least 95% for most vaccines. Achieving this target means contributing to the elimination, control, and, in some cases, such as smallpox, the eradication of vaccine-preventable diseases. However, in recent years, VC has shown a significant decline worldwide and also in Brazil, especially for the vaccines that make up the basic childhood immunization schedule^1^.

This context required the implementation of new strategies capable of improving VC. Thus, in Brazil, the Ministry of Health (MS), in 2023, reaffirming the National Movement for Vaccination established by the Brazilian government, began the microplanning process throughout the entire country as part of High-Quality Vaccination (AVAQ) activities. In microplanning, vaccination strategies are planned at the local level and are then expanded to broader levels, up to the national level^3^.

Through its practices, microplanning aims at the management, control, and elimination of various vaccine-preventable diseases (such as poliomyelitis, measles, and rubella), by applying criteria and indicators (effectiveness, homogeneity, timeliness, simultaneity, and efficiency) and identifying the socioeconomic, demographic, and social characteristics of each municipality. It also takes into account the catchment areas of basic health units (BHU), as well as the Family Health Strategy (FHS) teams that serve these locations^3^.

Such a reduction in VC, especially regarding the vaccines that make up the childhood immunization schedule, is multifactorial^1^, and is intensified by the phenomenon of vaccine hesitancy. This behavior is defined by the WHO as the intentional delay or refusal of recommended vaccines, regardless of whether they are available in health services or not. Furthermore, there is the additional complexity of encompassing the sociocultural and economic aspects of each social group, as well as the cultural, geographical, and financial characteristics of the states and municipalities involved ^1,4^. Thus, identifying the areas most prone to the transmission of vaccine-preventable diseases, as well as identifying the respective VC and carrying out risk classification for the transmission of these diseases, constitutes a significant strategy for establishing priorities and public actions, thereby enabling the control of these diseases^5,6^.

Thus, by analyzing the different characteristics of the municipalities, it is possible to infer that each locality has a certain profile of adequacy or inadequacy in terms of VC, as well as its own reasons and implications. Added to this, therefore, is the importance of carrying out risk classification for the transmission of vaccine-preventable diseases, as well as the possibility of implementing strategies and control measures focused on the prevention and management of such diseases and conditions^6^.

The aim of this study was to analyze the risk classification for the transmission of vaccine-preventable diseases in the municipalities of the state of São Paulo, as well as to assess the influence of population size on the risk of transmission of immunopreventable diseases before and after the implementation of microplanning for High-Quality Vaccination (AVAQ).

## METHODS

This is an epidemiological study with an ecological design, conducted using secondary data on vaccination coverage and dropout rates for nine immunobiologicals recommended in the vaccination schedule for children under two years of age in the state of São Paulo. It should be noted that the measles-mumps-rubella (MMR) vaccine was analyzed by considering the first and second doses separately. The period analyzed covered the years 2022 and 2023.

The state of São Paulo is composed of 645 municipalities, spread over 248,219 km² and, in 2022, had approximately 44,411,238 inhabitants. This federative unit is made up of 17 Regional Health Departments (RHD), which are responsible for coordinating the activities of the State Health Department at the regional level and promoting intersectoral collaboration^8^.

The municipalities of the state were classified according to population size, based on the previous study conducted by Braz et al.^5^, as follows: small-sized, when the estimated population was equal to or less than 20,000 inhabitants; medium-sized, with a population between 20,001 and 100,000 inhabitants; and large-sized, when it was greater than or equal to 100,001 inhabitants. It is worth noting that, in the state of São Paulo, 59.53% of the municipalities had an estimated population equal to or less than 20,000 inhabitants.

The selected immunobiologicals were: oral rotavirus vaccine (considering both the dose offered by the Unified Health System [SUS] and that offered by the private sector [pentavalent rotavirus]); meningococcal C vaccine (also considering the second dose of the meningococcal ACWY); pneumococcal vaccine (including the second dose of the 13-valent pneumococcal vaccine); pentavalent vaccine (including the third dose of the pentavalent vaccine and the third dose of the hexavalent vaccine in the private sector); poliomyelitis vaccine (also considering the third dose of the inactivated poliovirus vaccine [IPV] and the oral poliovirus vaccine [OPV], together with the inactivated pentavalent [DTaP+Hib+IPV] and the hexavalent); first dose of the measles-mumps-rubella (MMR) vaccine (including the first dose of the MMR-varicella and tetravalent vaccines); second dose of the MMR vaccine (considering the second dose of the MMR-varicella and the second dose or single dose of the tetravalent vaccine); yellow fever vaccine (considering single, initial, or first doses); first dose of the hepatitis A vaccine; and first dose of the varicella vaccine (also including the first dose or single dose of the tetravalent vaccine).

The doses of each immunobiological were obtained through the National Immunization Program Information System (SI-PNI), along with the data collected from the Ministry of Health website, in the Ministry of Health Monitoring Panel, filtered by place of residence. The BCG and hepatitis B vaccines were not analyzed, since in the state of São Paulo they are usually administered up to the time of hospital discharge of newborns, which could affect the analyses. It should be noted that, in one of the municipalities analyzed, for the year 2022, there were data only for the second dose of the meningococcal C vaccine; therefore, it was not possible to calculate the dropout rate for this immunobiological.

Vaccination coverage data for 2022 were extracted via Tabnet/Department of Informatics of the SUS (DATASUS)^a^, and for 2023 via the Vaccination Coverage Panel for the vaccines included in the National Immunization Schedule^b^. Both extractions were carried out on October 10, 2024, by place of residence. It should be noted that, starting in 2023, the Ministry of Health changed the way official information on vaccination in Brazil is made available. Until 2022, the data were accessed through Tabnet/DATASUS. From 2023 onward, queries began to be carried out in the Vaccination Coverage Panel of LocalizaSUS.

Subsequently, VC was organized according to the targets recommended by the PNI, namely greater than or equal to 90% for the human rotavirus vaccine and greater than or equal to 95% for the remaining immunobiologicals, resulting in the following categorization: very low (0% to less than 50%), low (greater than or equal to 50% and less than the target), and adequate (greater than or equal to the target and less than 120%).

Homogeneity among the vaccines followed the definition established by the study by Braz et al.^5^, and was therefore assessed by the proportion of immunobiologicals that reached the coverage targets in each municipality, in addition to the Organizational Contract for Public Health Action agreed upon by the SUS. It was classified as: adequate (greater than or equal to 70% and less than 100% of the nine vaccines whose VC was adequate — less than or equal to the target), low (greater than or equal to 50% and less than 70% of the nine vaccines with adequate coverage), and very low (less than 50% for the nine vaccines analyzed with adequate VC). The dropout rate was analyzed only for those vaccines with multidose schedules, such as the meningococcal C, pentavalent, 10-valent pneumococcal, poliomyelitis, and human rotavirus vaccines. For this purpose, the following calculation was used:


$$Dropoutrate=\frac{numberofindividualswhoreceivedthe1stdoseofthevaccin\text{e }}{numberofindividualswho\text{ r}eceivedthelastdoseofthevaccinationschedule}\times 100$$

After the calculation, dropout rates were classified as low (less than 5%), medium (greater than or equal to 5% and less than or equal to 10%), and high (greater than 10%). The municipalities also received a classification according to the RTDI indicator, available in the Health Surveillance Guide^9^ and according to the risk of transmission of vaccine-preventable diseases, as described in Braz et al.^5^ and adapted (Chart).

**Chart.** Synthesis of the parameters used for calculating the immunization indicators in this study, São Paulo, Brazil.

Risk indicator for the transmission of vaccine-preventable diseases	Low risk, when the homogeneity of vaccination coverage for the vaccines in the calendar for children aged ≤ 1 year is equal to 100%. Medium risk, when the homogeneity of vaccination coverage for the vaccines in the calendar for children aged ≤ 1 year "fluctuates" between ≥ 75 and < 100%. High risk, when the homogeneity of vaccination coverage for the vaccines in the calendar for children aged ≤ 1 year is < 75%
Braz et al.^5^ adapted^a^	Very low — municipality with vaccination coverage homogeneity (VCH) equal to 100%. Low — municipality with VCH ≥ 70% and less than 100%, with adequate vaccination coverage (VC) for the vaccines against poliomyelitis, measles, mumps, and rubella (MMR), and pentavalent. Medium — municipality with HCV ≥ 70% and < 100%, with CV below the target for up to two vaccines (poliomyelitis, MMR, or pentavalent). High — municipality with HCV < 70% (regardless of VC). Very high — municipality with VCH < 70%, a high dropout rate (> 10%) for any of the ten evaluated immunobiologicals, and a large population size and/or municipalities without vaccination records (any vaccine), regardless of population size

a Adaptation VCH ≤ 70%.

The data were analyzed using R software, version 4.4.1. Dropout rates were proportionally compared with vaccination coverage homogeneity (VCH) and RTDI classification according to the municipal population size using Pearson's χ^2^ test or Fisher's exact test, considering each year of analysis. To verify whether there was a significant reduction in the risk classifications for the reintroduction of vaccine-preventable diseases in the state of São Paulo between 2022 and 2023, the paired Wilcoxon signed-rank test was used. A 5% significance level was adopted for all analytical procedures.

Choropleth maps were created to examine the spatial distribution of the risk classification for the transmission of vaccine-preventable diseases in the state of São Paulo, by municipality. For this analytical procedure, the QGIS software, version 2.18.14, was used.

For this study, publicly available data without individual identification were used, thus waiving the need for approval by a research ethics committee.

## RESULTS

**Figure 1:**
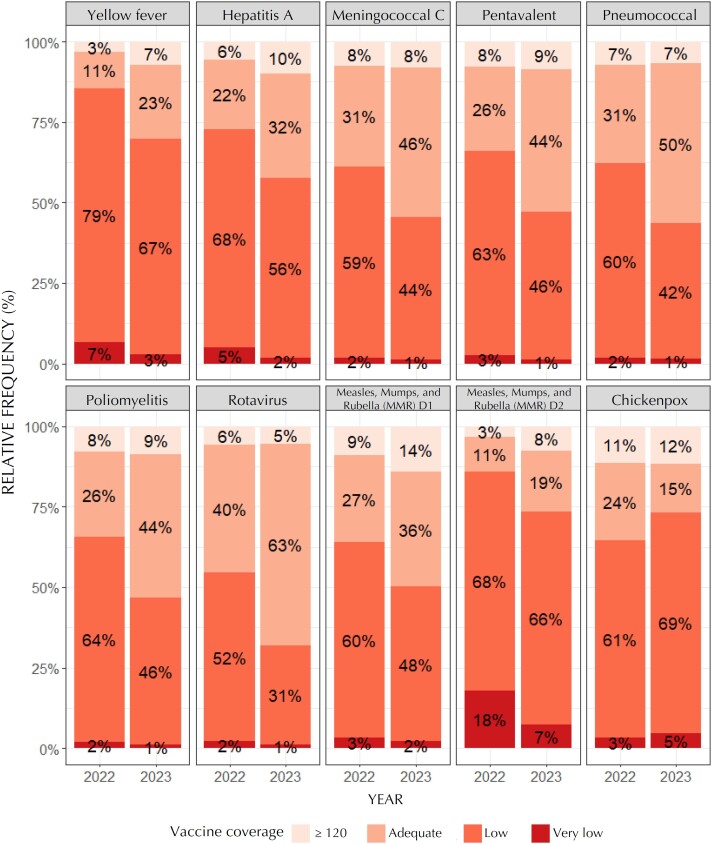
Percentage of municipalities according to the vaccine coverage classification, São Paulo, Brazil, 2022 and 2023^a^.

^a^645 municipalities; goal ≥ 90 for the human rotavírus vaccine; and ≥ 95 for the other immunobiologicals.

In 2022, among the nine immunobiologicals analyzed in the state of São Paulo, the human rotavirus vaccine showed the highest proportion (46%) of municipalities that reached the recommended coverage target, followed by the meningococcal vaccine (39%). Conversely, in the same year, 18% of the municipalities in the state of São Paulo had very low VC for the second dose of the MMR vaccine (Figure 1). In analyzing 2023, an increase was observed in the number of municipalities that reached the recommended target for eight of the nine vaccines analyzed, with the largest increase seen for the yellow fever vaccine (a 109% increase), followed by the second dose of the MMR vaccine (an 88% increase) and the hepatitis A vaccine (a 56% increase). Only the varicella vaccine showed reduction in the number of municipalities that achieved the recommended target, with a 24% decrease compared to 2022 (Figure 1).

When analyzing the dropout rate, VCH, and risk classification for the reintroduction of vaccine-preventable diseases according to municipal population size for the years 2022 and 2023, it was observed that small municipalities showed a higher proportion of low dropout compared with medium- and large-sized municipalities for the meningococcal C, 10-valent pneumococcal, poliomyelitis, and human rotavirus vaccines, with a statistically significant difference.

With regard to VCH, small municipalities in the state of São Paulo showed a higher proportion of VCH among vaccines with adequate coverage (≥ 70% to < 100%) compared with medium- and large-sized municipalities. Finally, in terms of the risk classification for the reintroduction of vaccine-preventable diseases, small municipalities were less frequently classified in the high- and very-high-risk categories compared with medium- and large-sized municipalities, considering both RTDI classifications and the classification by Braz et al.^5^ (Table 1).

**Table 1:** Dropout rates, homogeneity of vaccination coverage, and risk classification for the transmission of vaccine-preventable diseases according to the population size of the municipality, São Paulo, 2022 e 2023.

Indicator		Classification	Population size							
			2022				2023			
			Small	Medium	Large	P value	Small	Medium	Large	P value
			n (%)	n (%)	n (%)		n (%)	n (%)	n (%)	
Dropout rate	Meningococcal C	Low	249 (63.8)	121 (69.5)	61 (76.2)	< 0.001	231 (59.1)	103 (59.2)	57 (71.2)	< 0.001
		Medium	51 (13.1)	41 (23.6)	14 (17.5)		49 (12.5)	45 (25.9)	14 (17.5)	
		High	90 (23.1)	12 (6.9)	5 (6.2)		111 (28.4)	26 (14.9)	9 (11.2)	
	Pentavalent	Low	243 (62.1)	126 (72.4)	65 (81.2)	< 0.001	259 (66.2)	136 (78.2)	67 (83.8)	< 0.001
		Medium	65 (16.6)	32 (18.4)	10 (12.5)		49 (12.5)	24 (13.8)	8 (10.0)	
		High	83 (21.2)	16 (9.2)	5 (6.2)		83 (21.2)	14 (8.0)	5 (6.2)	
	Pneumococcal	Low	276 (70.6)	141 (81)	75 (93.8)	< 0.001	282 (72.1)	148 (85.1)	75 (93.8)	< 0.001
		Medium	57 (14.6)	27 (15.5)	5 (6.2)		61 (15.6)	21 (12.1)	4 (5.0)	
		High	58 (14.8)	6 (3.4)	0 (0)		48 (12.3)	5 (2.9)	1 (1.2)	
	Poliomyelitis	Low	244 (62.4)	117 (67.2)	64 (80)	< 0.001	256 (65.5)	135 (77.6)	70 (87.5)	< 0.001
		Medium	60 (15.3)	39 (22.4)	12 (15)		58 (14.8)	26 (14.9)	7 (8.8)	
		High	87 (22.3)	18 (10.3)	4 (5)		77 (19.7)	13 (7.5)	3 (3.8)	
	Rotavirus	Low	244 (62.4)	134 (77)	69 (86.2)	< 0.001	285 (72.9)	145 (83.3)	73 (91.2)	< 0.001
		Medium	83 (21.2)	31 (17.8)	10 (12.5)		62 (15.9)	24 (13.8)	7 (8.8)	
		High	64 (16.4)	9 (5.2)	1 (1.2)		44 (11.3)	5 (2.9)	0 (0)	
Homogeneity for < age 2		Adequate	132 (33.8)	10 (5.7)	0 (0)	< 0.001	166 (42.5)	49 (28.2)	4 (5.0)	< 0.001
		Low	53 (13.6)	23 (13.2)	2 (2.5)		83 (21.2)	31 (17.8)	8 (10.0)	
		Very low	206 (52.7)	141 (81)	78 (97.5)		142 (36.3)	94 (54)	68 (85.0)	
RTDI^9^ for ≤ age 1		Low	72 (18.4)	0 (0)	0 (0)	< 0.001	106 (27.1)	26 (14.9)	1 (1.2)	< 0.001
		Medium	52 (13.3)	16 (9.2)	0 (0)		66 (16.9)	32 (18.4)	6 (7.5)	
		High	267 (68.3)	158 (90.8)	80 (100)		219 (56)	116 (66.7)	73 (91.2)	
Braz et al.^5^ for younger than 2 years		Low and very low	57 (14.6)	1 (0.6)	0 (0)	< 0.001	106 (27.1)	22 (12.6)	2 (2.5)	< 0.001
		Medium	75 (19.2)	9 (5.2)	0 (0)		60 (15.3)	27 (15.5)	2 (2.5)	
		High and very high	259 (66.2)	164 (94.3)	80 (100)		225 (57.5)	125 (71.8)	76 (95.0)	

VCH: vaccination coverage homogeneity; RTDI: risk of transmission of vaccine-preventable diseases.

**Figure 2:**
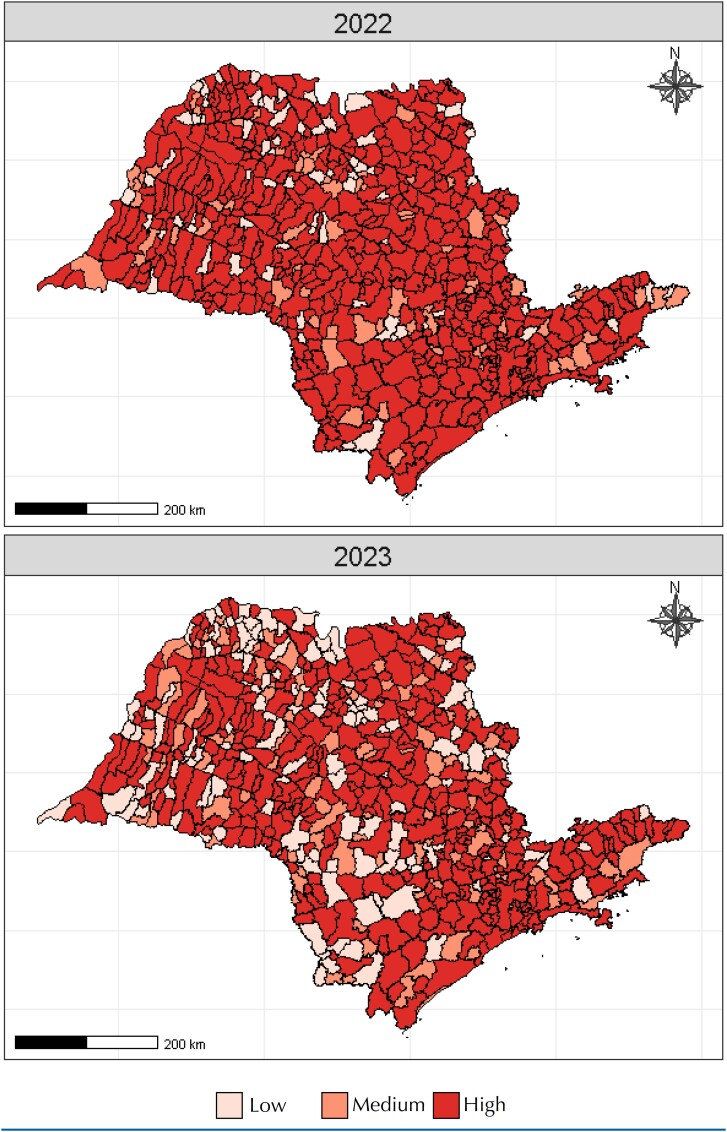
Spatial distribution of municipalities in the state of São Paulo according to the risk indicator for the transmission of vaccine-preventable diseases. São Paulo, Brazil, 2022 and 2023.

**Figure 3:**
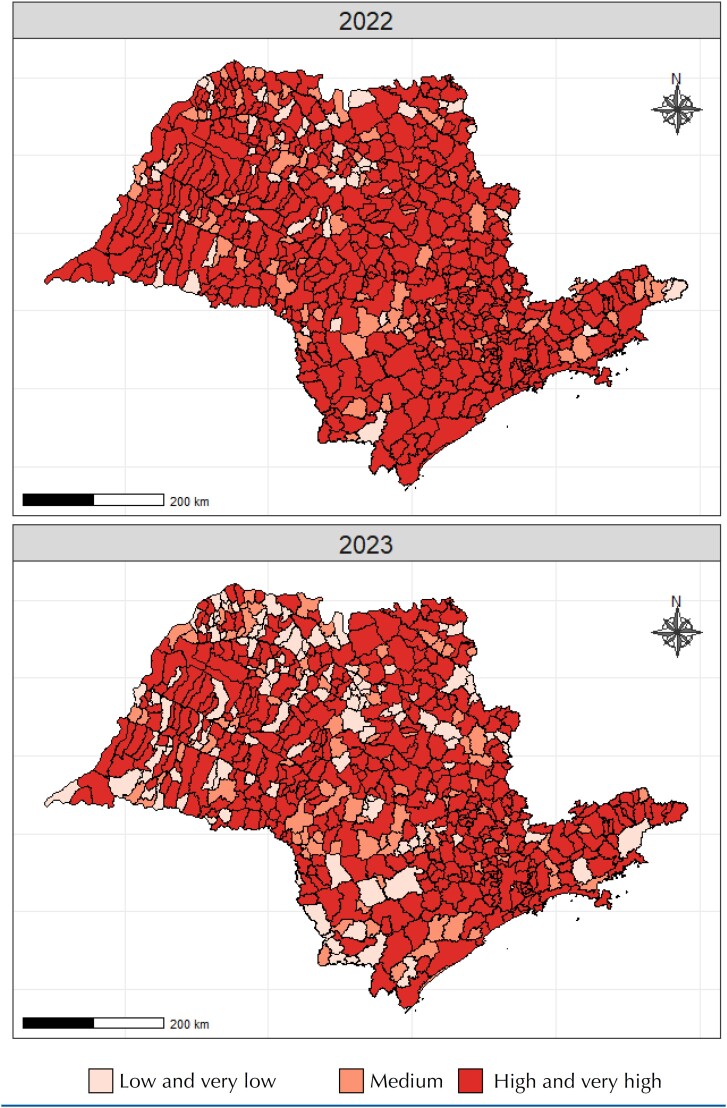
Spatial distribution of municipalities in the state of São Paulo according to classification regarding the risk of transmission of vaccine-preventable diseases, as described in Braz et al.^5^ and adapted. São Paulo, Brazil, 2022 and 2023.

In Table 2, the risk classification for the reintroduction of vaccine-preventable diseases in the state of São Paulo in 2022 and 2023 was examined according to the RTDI^9^ and the classification by Braz et al.^5^ It was observed that, for both methods, the proportion of cases classified as "low" risk in the RTDI^9^ classification and "very low and low" in the Braz et al.^5^ classification increased from 11.2% in 2022 to 20.6% and from 9% to 20.2% in 2023, respectively. The proportion of cases classified as "high" risk according to the RTDI^9^ and "high and very high" according to Braz et al.^5^ decreased from 78.3% in 2022 to 63.3% in 2023 and from 78% in 2022 to 66% in 2023, respectively, with a statistically significant difference (p < 0.001) (Table 2 and Figures 2 and 3).

**Table 2:** Risk classification for the reintroduction of vaccine-preventable diseases by year, São Paulo, 2022 and 2023, for children under two years of age.

		Years		P value
		2022	2023	
		n (%)	n (%)	
Risk classification (RTDI)^9^ for ≤ age1				
	Low	72 (11.2)	133 (20.6)	< 0.001
	Medium	68 (10.5)	104 (16.1)	
	High	505 (78.3)	408 (63.3)	
Risk classification (Braz et al.^5^) for younger than 2 years				
	Very low and low	58 (9.0)	130 (20.2)	< 0.001
	Medium	84 (13.0)	89 (13.8)	
	High and very high	503 (78.0)	426 (66.0)	

RTDI: risk of transmission of vaccine-preventable diseases.

## DISCUSSION

The analyses of vaccination coverage indicators, dropout rate, VCH, and risk classification for the reintroduction of vaccine-preventable diseases in the state of São Paulo for the years 2022 and 2023 showed that, in 2022, the human rotavirus vaccine had the highest proportion of municipalities that reached the coverage target, with 46%, followed by the meningococcal vaccine (38%).

In 2023, there was an increase in the number of municipalities that reached the target for eight of the nine vaccines analyzed, with yellow fever standing out, showing a 109% increase. Comparing 2022 and 2023, there was an overall improvement in vaccination coverage and a reduction in the risk of reintroduction of vaccine-preventable diseases, with an increase in the proportion of municipalities at "low" risk according to the RTDI^9^ classification and "very low and low" risk and a reduction in "high and very high" risk according to the classification by Braz et al.^5^ It is worth noting that, from the second half of 2023 onward, there was a significant shortage of the varicella vaccine, which may have affected the results.

Since its inception, the PNI has implemented effective strategies that reflect the guidelines of the WHO, achieving successes in expanding VC and, through this, enabling the eradication of vaccine-preventable diseases, as well as significantly reducing the occurrence of diseases such as diphtheria and pertussis. However, despite efforts to adhere to current vaccination schedules, the anti-vaccination movement has taken on an even greater scale and has had significant repercussions on the internet. During the Covid-19 pandemic, this movement intensified, resulting in a sharp and significant decline in vaccination rates, especially among the child population, considerably impacting public health safety^4,11,12^. Moreover, the risk of contagion from the virus, social isolation, and the limited movement of people in environments greatly reduced the demand for health services by the population, which harmed vaccination coverage for various vaccines^12^. The population, motivated by the fear of exposing children to the Covid-19 virus, avoided vaccination rooms even though the recommendations were to maintain immunizations^12^. The isolation, combined with the reorganization of primary care to address Covid-19 cases, resulted in a historic decline in the demand for vaccines, culminating in several delayed vaccination schedules worldwide^12^.

The reduction in infant mortality over the years is due to successful immunization programs^10,13^, thus the declines in vaccination coverage in the population, especially among children, is concerning^14^. Although Brazil was certified as a region free from the circulation of the measles virus by the Pan American Health Organization in 2016, the vaccination coverage rates for the measles, mumps, and rubella (MMR)^15-17^ vaccine have experienced a significant decline since then. In light of such an epidemiological reality in Brazil, measles was reintroduced and spread across its territory, making the country a risk area for the circulation of the virus in 2019, following the confirmation of sustained transmission of the disease in the country^15-17^.

In 2024, Brazil was recertified as a country free from measles. This result was achieved, among other initiatives, through the intensification of vaccination campaigns in border areas and hard-to-reach locations, active case finding, and the implementation of the "Day S" campaign to combat measles^18^.

Despite the recertification, the importance of continuing to develop tools with the potential to address the deficiency of information about immunobiologicals is emphasized, since misinformation combined with concerns about adverse reactions and the safety of vaccine administration are the main factors contributing to vaccine hesitancy^19^.

Regarding the risk classification, there was an increase in the number of cities classified as "very low and low" risk compared to 2023, and a reduction in cities classified as "high and very high" risk, more pronounced in small municipalities. A study conducted in the state of Minas Gerais observed the same phenomenon of the influence of population size on the indicators and the risk classification^6^. In this context, the role of the FHS is highlighted in ensuring equity in care, increasing vaccination coverage, and reducing infant mortality^20^.

It is evident that the implementation of the PNI occurs throughout Brazilian territory and is provided free of charge in primary health care (PHC) services. Historically, lower coverage of PHC is observed in more developed areas and among higher economic classes, and the deployment of the FHS in large urban centers is a challenge^21^. In the state of São Paulo, it was found that PHC coverage increased until 2016 and stabilized in the following years^22^.

Regarding physical availability and geographical accessibility, detailed studies on the reality of the BHU in Brazil are still scarce. However, it is possible to observe that there are differences in the quality of vaccination services between BHU, such as the availability of immunobiologicals and the presence of qualified professionals. This inequality often results in patients being referred to BHU located far from their homes, which can increase vaccination dropout^23^.

A notable example of a strategy from the PNI that remains to this day is the creation of the character *Zé Gotinha* in 1986, aimed at fostering closeness and trust between the population and the campaign against polio, through the sharing of technical and scientific information in a more dynamic and playful language. The mascot played an important role in increasing VC rates, especially between 2000 and 2015^24^. In addition, with a global focus, the Vaccine Confidence Project was established in 2010, with the objectives of early detection of potential vaccine hesitancy through dialogue with populations and relevant organizations, thus seeking to prevent gaps and dropouts in vaccination schedules^25^.

Adding to this scenario is the important role of microplanning, which demonstrates a positive impact on routine vaccination and the strengthening of the PNI in Brazil^3^. Furthermore, microplanning for the AVAQ, together with the decentralized multi-vaccination method, contributed to the increase in the number of vaccine doses administered in Brazil, the rise in vaccination coverage, and the number of municipalities and states that achieved the vaccination coverage target for most vaccines^3^.

Regarding the risk classification for the reintroduction of vaccine-preventable diseases, the results obtained, both by RTDI^9^ and by Braz et al.^5^, showed an improvement in the number of municipalities in the state of São Paulo classified with very low and low risk of reintroduction of vaccine-preventable diseases in 2023, compared to 2022.

Finally, it is important to note that this study has some limitations, particularly the use of secondary data sources. Thus, the researchers had no control over the quality of the recorded doses administered in the information systems, and they used different population bases as denominators for calculating VC. As mentioned, the monitoring panels of the PNI (Tabnet and LocalizaSUS) are a reliable method of assessment, consistently and effectively used to discuss health strategies and policies through vaccination coverage in the country^15^.

However, the use of rigorous methodology in this study is reinforced, which enables the monitoring of vaccination coverage and the repositioning of priority policies and strategies in municipalities with a risk of transmission of vaccine-preventable diseases. Thus, the study directly contributes to the improvement and success of the PNI in these areas, resulting in enhanced outbreak prevention and greater protection of public health^5^.

This study highlighted the increase in the number of municipalities that reached the recommended targets for most of the analyzed immunobiologicals. Regarding population size, small municipalities showed better indicators for dropout rates and adequate vaccination coverage and were classified in smaller proportions in the high and very high risk categories for the reintroduction of vaccine-preventable diseases compared to medium and large municipalities.

## Data Availability

The data are available at Tabnet/Datasus at http://tabnet.datasus.gov.br, and for 2023 via Vaccination Coverage Panel for the vaccines included in the National Calendar at https://infoms.saude.gov.br/extensions/seidigi_demas_vacinacao_calendario_nacional_cobertura_residencia/seidigi_demas_vacinacao_calendario_nacional_cobertura_residencia.html

## Footnotes

a Available from: http://tabnet.datasus.gov.br/cgi/dhdat.exe?bd_pni/cpnibr.def

b Available from: https://infoms.saude.gov.br/extensions/SEIDIGI_DEMAS_VACINACAO_CALENDARIO_NACIONAL_COBERTURA_RESIDENCIA/SEIDIGI_DEMAS_VACINACAO_CALENDARIO_NACIONAL_COBERTURA_RESIDENCIA.html
