# Supplementary material for: Risk of reintroduction of vaccine-preventable diseases in the state of São Paulo, Brazil
Source: Rev Saude Publica. 2026 Feb 23;60:e7. doi: 10.11606/s1518-8787.2026060007062 (PMC12928189; doi:10.11606/s1518-8787.2026060007062)
Supplement: Translation

# Translation: Risco de reintrodução de doenças preveníveis por vacinação no estado de São Paulo, Brasil

**Authors:** José Elisomar Silva de Santana, Maria Lígia Bacciotte Ramos Nerger, Tatiana Lang D’ Agostini, Joyce Ribeiro da Conceição Santos, Sheila Aparecida Ferreira Lachtim, Camilla Stephane Oliveira Silva, Thales Philipe Rodrigues da Silva, Fernanda Penido Matozinhos

**Affiliations:** I Coordenadoria de Controle de Doenças. Centro de Vigilância Epidemiológica "Prof. Alexandre Vranjac". Divisão de Imunização. São Paulo, SP, Brasil; II Universidade Federal de São Paulo. Escola Paulista de Enfermagem. São Paulo, SP, Brasil; III Universidade de São Paulo. Escola de Enfermagem. São Paulo, SP, Brasil; IV Universidade Federal de Minas Gerais. Escola de Enfermagem. Belo Horizonte, MG, Brasil; V Universidade Federal de Minas Gerais. Escola de Enfermagem. Departamento de Enfermagem Materno-Infantil e Saúde Pública. Belo Horizonte, MG, Brasil

**Keywords:** Cobertura Vacinal, Gestão de Risco, Monitoramento Ambiental, Vigilância em Desastres

## Abstract

**OBJETIVO:**

Analisar a classificação de risco de transmissão de doenças imunopreveníveis por vacinação nos municípios do estado de São Paulo (SP) e verificar a influência do porte populacional no risco de transmissão de doenças imunopreveníveis (RTDI) antes e depois da implementação do Microplanejamento para as Atividades de Vacinação de Alta Qualidade.

**MÉTODOS:**

Estudo epidemiológico, com delineamento ecológico, realizado com dados secundários de cobertura vacinal de nove imunobiológicos recomendados para o calendário vacinal de crianças menores de dois anos e da taxa de abandono para as vacinas de multidoses na mesma faixa etária no estado de SP, abrangendo os anos de 2022 e 2023. Após o cálculo de todos os indicadores, os municípios foram classificados conforme o risco de transmissão de doenças imunopreveníveis por vacinação.

**RESULTADOS:**

As análises dos indicadores de cobertura vacinal, taxa de abandono, homogeneidade de cobertura vacinal entre vacinas em menores de dois anos e indicador RTDI para menores ou igual a um ano revelaram que, em 2022, a vacina contra rotavírus humano apresentou a maior proporção de municípios que atingiram a meta de cobertura, com 46%, seguida pela vacina meningocócica (39%). No ano de 2023, a vacina contra rotavírus humano manteve-se com a maior proporção de municípios que atingiram a meta de cobertura (68%), seguida pela vacina pneumocócica (57%). Com relação ao RTDI, nos municípios de grande porte, o risco alto caiu de 100% para 91,2%, enquanto em municípios de médio porte houve redução de 90,8 para 66,7% e, nos de pequeno porte, de 68,3 para 56%.

**CONCLUSÃO:**

Existem desafios no estado quanto à classificação de RTDI por vacinação, especialmente em municípios de médio e grande porte. A utilização de indicadores de imunização por municípios pode ser bastante válida para a proposição de intervenções em saúde, visando ao aumento das coberturas vacinais.

## INTRODUÇÃO

O Programa Nacional de Imunizações (PNI) do Brasil, criado em 1973, é responsável pela operacionalização da Política Nacional de Imunizações e possui o objetivo de reduzir, controlar e eliminar a transmissão de doenças preveníveis por ação de vacinação. No cenário internacional, o PNI do Brasil é reconhecido mundialmente por seu amplo calendário vacinal, disponível para todos os ciclos de vida, e por reduzir a incidência e mortalidade de doenças preveníveis por ação de vacinação, além da erradicação de algumas delas^1,2^. Com relação ao histórico do Programa Estadual de Imunização de São Paulo, as ações de imunização no estado tiveram atividades registradas desde 1962, previamente à criação do PNI, quando ocorreu a primeira campanha estadual de vacinação contra a poliomielite. No ano de 1968, houve a publicação da primeira Norma Técnica do Programa de Imunização da Secretaria de Estado da Saúde de São Paulo.

A Organização Mundial da Saúde (OMS) ressalta que as taxas de cobertura vacinal (CV) devem ser de pelo menos 95% para a maioria das vacinas. Atingir essa meta significa contribuir para a eliminação, o controle e, em alguns casos, como no da varíola, a erradicação de doenças preveníveis por ação de vacinação. Entretanto, nos últimos anos, a CV apresentou queda significativa no mundo e também no Brasil, principalmente para as vacinas que compõem o calendário básico infantil^1^.

Tal contexto exigiu a implementação de novas estratégias capazes de melhorar as CV. Desta forma, no Brasil, o Ministério da Saúde (MS), no ano de 2023, ratificando o Movimento Nacional pela Vacinação instituído pelo governo brasileiro, iniciou em todo o território brasileiro o processo de microplanejamento, como parte das atividades de vacinação de alta qualidade (AVAQ). No microplanejamento, as estratégias de vacinação são planejadas desde o nível local e são ampliadas para os níveis mais amplos, até o nível nacional^3^.

O microplanejamento, por meio de suas práticas, visa ao manejo, controle e eliminação de diversas doenças preveníveis por ação de vacinação (como poliomielite, sarampo e rubéola), com a aplicação de critérios e indicadores (eficácia, homogeneidade, oportunidade, simultaneidade e eficiência) e identificação das características socioeconômicas, demográficas e sociais de cada município. Considera, também, as áreas de abrangência das unidades básicas de saúde (UBS), além das equipes de Estratégia Saúde da Família (ESF) que compõem essas localidades^3^.

A redução nas CV, especialmente com relação as vacinas que compõem o calendário infantil, é multicausal^1^, sendo potencializada pelo fenômeno da hesitação vacinal. Tal comportamento é definido pela OMS como o atraso proposital ou recusa das vacinas recomendadas, independentemente de estas estarem disponíveis ou não nos serviços de saúde. Soma-se, ainda, a complexidade de abarcar aspectos socioculturais e econômicos de cada grupo social, bem como características culturais, geográficas e financeiras dos estados e municípios envolvidos^1,4^. Assim, realizar a identificação das áreas mais propensas à transmissão de doenças preveníveis por ação de vacinação, além da identificação das respectivas CV e a realização de classificação de risco para transmissão dessas doenças, constitui-se em estratégia significativa para a construção de prioridades e ações públicas, oportunizando, assim, o controle dessas doenças^5,6^.

Sendo assim, ao analisar as diferentes características dos municípios, é possível depreender que cada localidade possui determinado perfil de adequação ou não à CV, bem como seus motivos e implicações. Soma-se a isso, portanto, a importância da realização da classificação de risco de transmissão de doenças preveníveis por ação de vacinação, bem como a possibilidade de implementação de estratégias e meios de controle, com foco na prevenção e manejo de tais doenças e agravos^6^.

O objetivo deste estudo foi analisar a classificação de risco de transmissão de doenças preveníveis por vacinação nos municípios do estado de São Paulo, bem como verificar a influência do porte populacional no risco de transmissão de doenças imunopreveníveis antes e depois da implementação do microplanejamento para as AVAQ.

## MÉTODOS

Trata-se de um estudo epidemiológico, com delineamento ecológico, realizado com dados secundários de CV e da taxa de abandono de nove imunobiológicos recomendados para o calendário vacinal de crianças menores de dois anos no estado de São Paulo. Ressalta-se que a vacina tríplice viral foi analisada considerando separadamente primeira e segunda dose. O período analisado compreendeu os anos de 2022 e 2023.

O estado de São Paulo é composto de 645 municípios, distribuídos em 248.219 km² e, em 2022, contava com aproximadamente 44.411.238 habitantes^7^. Essa unidade federativa é composta de 17 Departamentos Regionais de Saúde — DRS, cuja responsabilidade é coordenar as atividades da Secretaria de Estado da Saúde no âmbito regional e promover articulações^8^.

Os municípios do estado foram classificados segundo o porte populacional, baseando-se no estudo prévio realizado por Braz et al.^5^, como se segue: pequeno porte, quando a população estimada era igual ou menor a 20.000 habitantes; médio porte, com população entre 20.001 e 100.000 habitantes; e grande porte, quando maior ou igual a 100.001 habitantes. Vale ressaltar que, no estado de São Paulo, 59,53% dos municípios do estado apresentavam população estimada igual ou menor a 20.000 habitantes.

Os imunobiológicos selecionados foram: vacina oral contra o rotavírus (considerando-se tanto a dose ofertada pelo Sistema Único de Saúde [SUS] quanto a ofertada pela rede privada [rota-pentavalente]), vacina contra a doença meningocócica C (considerando também a segunda dose da meningocócica ACWY), vacina contra a doença pneumocócica (incluindo a segunda dose da pneumocócica 13V), vacina pentavalente (incluindo a terceira dose da vacina pentavalente e a terceira dose da vacina hexavalente na rede privada), vacina contra a poliomielite (considerando também a terceira dose da vacina inativada poliomielite [VIP] e vacina oral poliomielite [VOP], junto com a pentavalente inativada [DTPa+Hib+VIP] e a hexavalente), primeira dose da vacina tríplice viral (incluindo a primeira dose das vacinas quádrupla viral e tetraviral), segunda dose da vacina tríplice viral (considerando a segunda dose da quádrupla viral e segunda dose ou dose única da tetraviral), vacina contra a febre amarela (considerando aquelas de dose única, inicial ou primeira dose), primeira dose da vacina contra a hepatite A e primeira dose da vacina contra a varicela (incluindo também a primeira dose ou dose única da vacina tetraviral).

As doses de cada imunobiológico foram acessadas por meio do Sistema de Informações do Programa Nacional de Imunizações (SI-PNI), junto com os dados, que foram coletados do site do MS, no Painel de Monitoramento do Ministério da Saúde, filtrado por residência. As vacinas BCG e hepatite B não foram analisadas, pois no estado de São Paulo elas normalmente são administradas até o momento da alta hospitalar dos recém-nascidos, o que poderia causar alterações nas análises. Ressalta-se que, em um dos municípios analisados, para o ano de 2022, havia dados somente da segunda dose da vacina meningo C; sendo assim, não foi possível calcular a taxa de abandono para esse imunobiológico.

As CV foram extraídas, para o ano de 2022, via Tabnet/Departamento de Informática do SUS (DATASUS)^a^ e, para o ano de 2023, via Painel da Cobertura Vacinal das vacinas presentes no Calendário Nacional^b^. Ambas as extrações foram realizadas no dia 10 de outubro de 2024, por residência. Ressalta-se que, a partir de 2023, o MS alterou a forma de disponibilizar as informações oficiais sobre a vacinação no território brasileiro. Até o ano de 2022, os dados eram acessados por meio do Tabnet/DATASUS. A partir de então, desde 2023, as consultas passaram a ser realizadas no Painel de Cobertura Vacinal do LocalizaSUS.

Posteriormente, as CV foram organizadas segundo as metas preconizadas pelo PNI, como maior ou igual a 90% para a vacina contra o rotavírus humano e maior ou igual a 95% para o restante dos imunobiológicos, obtendo-se a seguinte categorização: muito baixa (0% a menor que 50%), baixa (maior ou igual a 50% e menor que a meta) e adequada (maior ou igual à meta a menor que 120%).

A homogeneidade entre as vacinas seguiu a definição estabelecida pelo estudo de Braz et al.^5^, sendo então avaliada pela proporção de imunobiológicos que atingiram as metas de cobertura em cada município, além do Contrato Organizativo de Ação Pública da Saúde acordado pelo SUS. Foi classificada em: adequada (maior ou igual a 70% a menor que 100% das nove vacinas cuja cobertura vacinal estava adequada — menor ou igual à meta), baixa (maior ou igual a 50% a menor que 70% das nove vacinas com cobertura adequada) e muito baixa (menor que 50% para as nove vacinas analisadas com cobertura vacinal adequada). A taxa de abandono foi analisada somente em relação àquelas vacinas com esquema multidoses, como vacina contra a doença meningocócica C, pentavalente, pneumocócica 10, poliomielite e rotavírus humano. Para este fim, adotou-se o seguinte cálculo:


$$\begin{array}{c} \text{ (}númerodeindivíduoquereceberama1^{a}dosedavacina\text{)- }\\ Taxadeabandono=\frac{\left(númerodeindivíduoquereceberamaúltimadosedoesquemavacinal\right)}{\left(númerodeindivíduoquereceberama1^{\text{a}}dosedavacina\right)}\times 100 \end{array}$$

Após o cálculo, as taxas de abandono foram classificadas em: baixa (menor que 5%), média (maior ou igual a 5% e menor ou igual a 10%) e alta (maior que 10%). Os municípios também receberam uma classificação quanto indicador de RTDI, disponível no Guia de Vigilância em Saúde^9^ e segundo classificação quanto ao risco de transmissão de doenças preveníveis por ação de vacinação, como descrito em Braz et al.^5^ e adaptado (Quadro).

**Quadro.** Síntese dos parâmetros utilizados para cálculo dos indicadores de imunização neste estudo, São Paulo, Brasil.

Indicador de risco de transmissão de doenças imunopreveníveis	Baixo risco, quando a homogeneidade de coberturas das vacinas do calendário das crianças com idade ≤ 1 ano for igual a 100%. Médio risco, quando a homogeneidade de coberturas das vacinas do calendário das crianças com idade ≤ 1 ano "flutuar" entre ≥ 75 e < 100%. Alto risco, quando a homogeneidade de coberturas das vacinas do calendário das crianças com idade ≤ 1 ano for < 75%.
Braz et al.^5^ adaptado^a^	Muito baixo — município com homogeneidade de cobertura vacinal (HCV) igual 100%. Baixo — município com HCV ≥ 70% e menor que < 100%, com cobertura vacinal (CV) adequada para as vacinas contra poliomielite, tríplice viral e pentavalente. Médio — município com HCV ≥ 70% e < 100%, com CV abaixo da meta para até duas vacinas (poliomielite, tríplice viral ou pentavalente). Alto — município com HCV < 70% (independente da CV). Muito alto — município com HCV < 70%, taxa de abandono alta (> 10%) para qualquer um dos dez imunobiológicos avaliados e com grande porte populacional e/ou municípios sem registro vacinal (qualquer vacina), independentemente do porte populacional.

a Adaptação: HCV ≤ 70%.

Os dados foram analisados por meio do *software* R, versão 4.4.1. As taxas de abandono foram comparadas proporcionalmente à homogeneidade de cobertura vacinal (HCV) e à classificação de RTDI segundo o porte populacional do município por meio do teste χ^2^ de Pearson ou exato de Fisher, considerando-se cada ano de análise. Para verificar se houve redução significativa nas classificações de risco para a reintrodução de doenças preveníveis por ação de vacinação no estado de São Paulo entre os anos de 2022 e 2023, utilizou-se o teste de postos sinalizados de Wilcoxon pareado. Adotou-se o nível de significância de 5% para o procedimento analítico em sua totalidade.

Foram construídos mapas coropléticos para verificar a distribuição espacial da classificação de risco para a transmissão de doenças preveníveis por ação de vacinação no estado de São Paulo, por município. Para esse procedimento analítico, utilizou-se o programa QGIS, versão 2.18.14.

Para a realização deste estudo, foram utilizados dados de domínio público e sem identificação individual, dispensando a aprovação de um comitê de ética e pesquisa.

## RESULTADOS

No ano de 2022, para os nove imunobiológicos analisados no estado de São Paulo, a vacina rotavírus humano apresentou a maior proporção (46%) de municípios que alcançaram a meta de cobertura preconizada, seguida da vacina meningocócica (39%). Em contrapartida, neste mesmo ano, 18% dos municípios do estado de São Paulo apresentaram a CV muito baixa para a segunda dose da vacina tríplice viral (Figura 1). Ao se analisar o ano de 2023, observou-se aumento do número de municípios que alcançaram a meta preconizada para oito das nove vacinas analisadas, sendo o maior aumento o de número de municípios que alcançaram a meta para a vacina contra febre amarela (aumento de 109%), seguida da vacina tríplice viral D2 (aumento de 88%) e vacina contra hepatite A (aumento de 56%). Somente para a vacina contra varicela se observou redução no número de municípios que atingiram a meta preconizada, de 24% em relação ao ano de 2022 (Figura 1).

**Figura 1:** Porcentagem de municípios segundo classificação de cobertura vacinal, São Paulo, Brasil, 2022 e 2023^a^ .

^a^645 municípios; meta ≥ 90 para a vacina rotavírus humano e ≥ 95 para os demais imunobiológicos.

Ao se analisar a taxa de abandono, HCV e classificação de risco para a reintrodução de doenças preveníveis por ação de vacinação segundo o porte populacional do município para o ano de 2022 e 2023, observou-se que municípios de pequeno porte apresentaram maior proporção de baixo abandono em comparação com municípios de médio e grande porte para as vacinas contra meningococo C, pneumo 10, poliomielite e rotavírus humano, com diferença estatisticamente significativa.

Com relação à HCV, municípios de pequeno porte do estado de São Paulo apresentaram maior proporção de HCV entre as vacinas adequadas (≥ 70 a < 100%) para os municípios de médio e grande porte. Por fim, quanto à classificação de risco para a reintrodução de doenças preveníveis por ação de vacinação, municípios de pequeno porte foram classificados em menor proporção na categoria de risco alto e muito alto em comparação com municípios de médio e grande porte, considerando-se as duas classificações RTDI^9^ e a classificação de Braz et al.^5^ (Tabela 1).

**Tabela 1:** Taxa de abandono, homogeneidade de cobertura vacinal e classificação de risco para a transmissão de doenças preveníveis por vacinas segundo o porte populacional do município, São Paulo, 2022 e 2023.

Indicador		Classificação	Porte populacional							
			2022				2023			
			Pequeno	Médio	Grande	Valor p	Pequeno	Médio	Grande	Valor p
			n (%)	n (%)	n (%)		n (%)	n (%)	n (%)	
Taxa de abandono	Meningo C	Baixa	249 (63,8)	121 (69,5)	61 (76,2)	< 0,001	231 (59,1)	103 (59,2)	57 (71,2)	< 0,001
		Média	51 (13,1)	41 (23,6)	14 (17,5)		49 (12,5)	45 (25,9)	14 (17,5)	
		Alta	90 (23,1)	12 (6,9)	5 (6,2)		111 (28,4)	26 (14,9)	9 (11,2)	
	Pentavalente	Baixa	243 (62,1)	126 (72,4)	65 (81,2)	< 0,001	259 (66,2)	136 (78,2)	67 (83,8)	< 0,001
		Média	65 (16,6)	32 (18,4)	10 (12,5)		49 (12,5)	24 (13,8)	8 (10,0)	
		Alta	83 (21,2)	16 (9,2)	5 (6,2)		83 (21,2)	14 (8,0)	5 (6,2)	
	Pneumocócica	Baixa	276 (70,6)	141 (81)	75 (93,8)	< 0,001	282 (72,1)	148 (85,1)	75 (93,8)	< 0,001
		Média	57 (14,6)	27 (15,5)	5 (6,2)		61 (15,6)	21 (12,1)	4 (5,0)	
		Alta	58 (14,8)	6 (3,4)	0 (0)		48 (12,3)	5 (2,9)	1 (1,2)	
	Poliomielite	Baixa	244 (62,4)	117 (67,2)	64 (80)	< 0,001	256 (65,5)	135 (77,6)	70 (87,5)	< 0,001
		Média	60 (15,3)	39 (22,4)	12 (15)		58 (14,8)	26 (14,9)	7 (8,8)	
		Alta	87 (22,3)	18 (10,3)	4 (5)		77 (19,7)	13 (7,5)	3 (3,8)	
	Rotavírus	Baixa	244 (62,4)	134 (77)	69 (86,2)	< 0,001	285 (72,9)	145 (83,3)	73 (91,2)	< 0,001
		Média	83 (21,2)	31 (17,8)	10 (12,5)		62 (15,9)	24 (13,8)	7 (8,8)	
		Alta	64 (16,4)	9 (5,2)	1 (1,2)		44 (11,3)	5 (2,9)	0 (0)	
Homogeneidade para < 2 anos		Adequada	132 (33,8)	10 (5,7)	0 (0)	< 0,001	166 (42,5)	49 (28,2)	4 (5,0)	< 0,001
		Baixa	53 (13,6)	23 (13,2)	2 (2,5)		83 (21,2)	31 (17,8)	8 (10,0)	
		Muito baixa	206 (52,7)	141 (81)	78 (97,5)		142 (36,3)	94 (54)	68 (85,0)	
RTDI^9^ para ≤ 1 ano		Baixo	72 (18,4)	0 (0)	0 (0)	< 0,001	106 (27,1)	26 (14,9)	1 (1,2)	< 0,001
		Médio	52 (13,3)	16 (9,2)	0 (0)		66 (16,9)	32 (18,4)	6 (7,5)	
		Alto	267 (68,3)	158 (90,8)	80 (100)		219 (56)	116 (66,7)	73 (91,2)	
Braz et al.^5^ para menores de 2 anos		Baixo e muito baixo	57 (14,6)	1 (0,6)	0 (0)	< 0,001	106 (27,1)	22 (12,6)	2 (2,5)	< 0,001
		Médio	75 (19,2)	9 (5,2)	0 (0)		60 (15,3)	27 (15,5)	2 (2,5)	
		Alto e muito alto	259 (66,2)	164 (94,3)	80 (100)		225 (57,5)	125 (71,8)	76 (95,0)	

HCV: homogeneidade de cobertura vacinal; RTDI: indicador de risco de transmissão de doenças imunopreveníveis.

Na Tabela 2, verificou-se a classificação de risco para a reintrodução de doenças preveníveis por ação de vacinação no estado de São Paulo nos anos de 2022 e 2023, segundo o RTDI^9^ e a classificação de Braz et al.^5^ Observou-se que, para ambos os métodos, a proporção de casos classificados como de risco "baixo" na classificação RTDI^9^ e "muito baixo e baixo" na classificação de Braz et al.^5^ aumentou de 11,2% em 2022 para 20,6% e de 9% para 20,2% em 2023, respectivamente. A proporção de casos classificados como de risco "alto" segundo a RTDI^9^ e "alto e muito alto" segundo Braz et al.^5^ diminuiu de 78,3% em 2022 para 63,3% em 2023 e de 78% em 2022 para 66% em 2023, respectivamente, com diferença estatisticamente significativa (p < 0,001) (Tabela 2 e Figuras 2 e 3).

**Tabela 2:** Classificação de risco para a reintrodução de doenças preveníveis por vacinas segundo o ano, São Paulo, 2022 e 2023, para crianças menores de dois anos.

		Anos		Valor p
		2022	2023	
		n (%)	n (%)	
Classificação de risco (RTDI)^9^ para ≤ 1 ano				
	Baixo	72 (11,2)	133 (20,6)	< 0,001
	Médio	68 (10,5)	104 (16,1)	
	Alto	505 (78,3)	408 (63,3)	
Classificação de risco (Braz et al.^5^) para menores de 2 anos				
	Muito baixo e baixo	58 (9,0)	130 (20,2)	< 0,001
	Médio	84 (13,0)	89 (13,8)	
	Alto e muito alto	503 (78,0)	426 (66,0)	

RTDI: indicador de risco de transmissão de doenças imunopreveníveis.

**Figura 2:** Distribuição espacial dos municípios do estado de São Paulo segundo o indicador de risco de transmissão de doenças preveníveis por vacinas. São Paulo, Brasil, 2022 e 2023.

**Figura 3:** Distribuição espacial dos municípios do estado de São Paulo segundo classificação quanto ao risco de transmissão de doenças preveníveis por vacinas, como descrito em Braz et al.^5^ e adaptado. São Paulo, Brasil, 2022 e 2023.

## DISCUSSÃO

As análises dos indicadores de CV, taxa de abandono, HCV e classificação de risco para reintrodução de doenças preveníveis por ação de vacinação no estado de São Paulo, para os anos de 2022 e 2023, demonstraram que, em 2022, a vacina contra rotavírus humano foi a que apresentou a maior proporção de municípios do estado que atingiram a meta de cobertura, com 46%, seguida pela vacina meningocócica (38%).

Em 2023, observou-se aumento no número de municípios que alcançaram a meta para oito das nove vacinas analisadas, destacando-se a febre amarela, com aumento de 109%. Comparando-se os anos 2022 e 2023, houve melhora geral na CV e redução no risco de reintrodução de doenças preveníveis por ação de vacinação, com aumento na proporção de municípios em risco "baixo" pela classificação RTDI^9^ e "muito baixo e baixo" e redução no risco "alto e muito alto" pela classificação de Braz et al.^5^ Vale ressaltar que, a partir do segundo semestre de 2023, houve desabastecimento importante da vacina varicela, o que pode ter interferido no resultado.

Desde a sua criação, o PNI implementou estratégias eficazes que refletem as diretrizes da OMS, alcançando sucessos na ampliação da CV e, por meio desta, possibilitou a erradicação de doenças preveníveis por ação de vacinação, bem como a redução significativa na ocorrência de doenças, como difteria e coqueluche^10^. Contudo, apesar dos esforços para seguir os calendários vigentes, o movimento antivacinal tomou uma proporção ainda maior e importante repercussão na internet. Durante a pandemia de covid-19 esse movimento se intensificou^8^, o que resultou em queda abrupta e significativa nas taxas de CV, especialmente entre o público infantil, afetando consideravelmente a segurança sanitária^4,11,12^. Além disso, o risco do contágio pelo vírus, o isolamento social e a circulação limitada de pessoas nos ambientes reduziu grandemente a procura dos serviços de saúde pela população, o que prejudicou a cobertura vacinal de diversas vacinas^12^. A população, motivada pelo medo de expor as crianças ao vírus da covid-19, evitou as salas de vacinação mesmo que as recomendações de que as imunizações devessem ser mantidas^12^. O isolamento, alinhado com a reorganização da atenção primária para atender os casos de covid-19, resultou em uma queda histórica pela procura de vacinas, culminando em vários calendários vacinais atrasados no mundo^12^.

A redução da mortalidade infantil ao longo dos anos deve-se a programas de imunização bem-sucedidos^10,13^, de modo que as quedas das CV na população, especialmente das crianças, é preocupante^14^. Apesar o Brasil de ter sido certificado como área livre da circulação do vírus do sarampo pela Organização Pan-Americana da Saúde em 2016, as taxas de CV da vacina tríplice viral^15-17^ — que confere proteção contra sarampo, caxumba e rubéola — vêm sofrendo declínio acentuado desde então. Diante de tal realidade epidemiológica do Brasil, o sarampo foi reintroduzido e disseminado em seu território, tornando o país área de risco à circulação do vírus em 2019, após a confirmação de transmissão sustentada da doença no país^15-17^.

Em 2024, o Brasil foi recertificado como país livre do sarampo. Esse resultado foi alcançado, entre outras iniciativas, por meio da intensificação das campanhas de vacinação em áreas de fronteira e locais de difícil acesso, da busca ativa de casos suspeitos e da realização do Dia S de combate ao sarampo^18^.

Apesar da recertificação, ressalta-se a importância da continuidade de elaboração de instrumentos com potencial para combater a deficiência de informações acerca dos imunobiológicos, uma vez que a desinformação aliada a inquietações quanto às reações adversas e quanto à segurança na administração das vacinas são os principais fatores de hesitação vacinal^19^.

No que tange à classificação de risco, observou-se aumento das cidades classificadas como de risco "muito baixo e baixo" em relação ao ano de 2023 e redução para cidades classificadas como de risco "alto e muito alto", mais expressiva nos municípios de pequeno porte. Estudo realizado no estado de Minas Gerais observou o mesmo fenômeno da influência do porte populacional nos indicadores e na classificação de risco^6^. Nesse contexto, destaca-se o papel da ESF na garantia da equidade do cuidado, aumento das CV e redução da mortalidade infantil^20^.

É notório que a materialização do PNI ocorre em todo o território brasileiro e de forma gratuita nos serviços de atenção primária à saúde (APS). Observa-se menor cobertura da APS, historicamente, nos territórios mais desenvolvidos e nas classes econômicas mais elevadas, e que a implantação da ESF em grandes centros urbanos é um desafio^21^. No estado de São Paulo, constatou-se que cobertura da APS apresentou aumento até o ano de 2016 e estabilização nos anos seguintes^22^.

No que diz respeito à disponibilidade física e à acessibilidade geográfica, ainda são escassos os estudos detalhados sobre a realidade das UBS no Brasil. Entretanto, é possível observar que existem diferenças na qualidade do serviço de vacinação entre UBS, como disponibilidade dos imunobiológicos e presença de profissionais qualificados. Essa desigualdade resulta muitas vezes no encaminhamento dos pacientes para UBS distantes de suas residências, o que pode aumentar a desistência da vacinação^23^.

Um exemplo notável de estratégia do PNI, que permanece até os dias atuais, é a criação do personagem Zé Gotinha em 1986, que objetivou a aproximação e confiança da população na campanha contra a poliomielite, por meio de informações técnico-científicas compartilhadas em uma linguagem mais dinâmica e lúdica. O mascote desempenhou um papel importante no aumento das taxas de CV, especialmente entre os anos 2000 e 2015^24^. Além disso, com atuação global, em 2010 foi criado o *Vaccine Confidence Project*, cujos objetivos são detectar precocemente possíveis hesitações vacinais por meio de diálogos com as populações e os órgãos vigentes, buscando-se assim evitar lacunas e abandonos nos calendários vacinais^25^.

Soma-se a esse cenário o papel importante do microplanejamento, que demonstra impacto positivo na vacinação de rotina e no fortalecimento do PNI no Brasil^3^. Além disso, o microplanejamento para as AVAQ, em conjunto com o método de multivacinação descentralizada, contribuiu para o aumento do número de doses de vacinas aplicadas no Brasil, o aumento da CV e do número de municípios e estados brasileiros que alcançaram a meta da CV para a maioria das vacinas^3^.

Com relação à classificação de risco para a reintrodução de doenças preveníveis por ação de vacinação, os resultados obtidos, tanto pelo RTDI^9^ quanto por Braz et al.^5^, mostraram melhora na quantidade de municípios do estado de São Paulo que foram classificados com risco muito baixo e baixo de reintrodução de doenças preveníveis por ação de vacinação em 2023, em comparação com 2022.

Por fim, ressalta-se que este estudo apresenta algumas limitações, destacando-se o uso de bases de dados secundárias. Assim, os pesquisadores não tiveram controle sobre a qualidade do registro de doses aplicadas nos sistemas de informação, além de terem utilizado bases populacionais diferentes como denominador para o cálculo das CV. Como mencionado, os painéis de monitoramento do PNI (Tabnet e LocalizaSUS) são uma forma segura de apuração, consistente e bem utilizada para discutir estratégias e políticas de saúde por meio da cobertura vacinal no país^15^.

Todavia, reforça-se o uso de metodologia rigorosa neste estudo, que possibilita o acompanhamento das CV e a recolocação de políticas e estratégias prioritárias nos municípios com risco de transmissão de doenças preveníveis por ação de vacinação. Assim, o estudo contribui de maneira direta para o aprimoramento e êxito do PNI nesses locais, o que resulta em ampliação da prevenção de surtos e maior proteção da saúde da população^5^.

Este estudo ressaltou o aumento no número de municípios que atingiram as metas preconizadas para a maioria dos imunobiológicos analisados. Com relação ao porte populacional, municípios de pequeno porte apresentaram melhores indicadores para taxa de abandono e HCV adequada e foram classificados em menor proporção na categoria de risco alto e muito alto de reintrodução de doenças preveníveis por ação de vacinação em comparação com municípios de médio e grande porte.

## Data Availability

Os dados estão disponíveis via Tabnet/Datasus em http://tabnet.datasus.gov.br, e para o ano de 2023 via Painel da Cobertura Vacinal das vacinas presentes no Calendário Nacional em https://infoms.saude.gov.br/extensions/seidigi_demas_vacinacao_calendario_nacional_cobertura_residencia/seidigi_demas_vacinacao_calendario_nacional_cobertura_residencia.html

## Footnotes

a Disponível em: http://tabnet.datasus.gov.br/cgi/dhdat.exe?bd_pni/cpnibr.def

b Disponível em: https://infoms.saude.gov.br/extensions/SEIDIGI_DEMAS_VACINACAO_CALENDARIO_NACIONAL_COBERTURA_RESIDENCIA/SEIDIGI_DEMAS_VACINACAO_CALENDARIO_NACIONAL_COBERTURA_RESIDENCIA.html
